# In Re Itinerant Dental Instructors

**Published:** 1919-06

**Authors:** 


### IN RE ITINERANT DENTAL INSTRUCTORS

At a recent meeting of the Los Angeles County Dental Society a resolution was unanimously passed for the purpose of restraining, as far as it may be possible by such means, the ever-growing epidemic of itinerant dental instructors from which this and other communities have for years past been the uncomplaining victims. The society with a membership of around five hundred has gone on record as opposed to any form of graduate and post-graduate instruction which is not fostered by an educational institution, a dental society or a study club composed of members in good standing in their respective dental societies. The itinerant dispenser of so-called dental education is invited to remain as far away as possible from the sphere of influence of the Los Angeles County Dental Society.

The resolution in question reflects the state of mind of those who in the past have been prevailed upon to become members of classes organized for the purpose of improving technical methods or to broaden scientific foundations. These courses with hardly any exception at all have been started primarily for the lust of money of their promoters or to further the sale of some dental product, an instance of the latter variety being still vivid in the minds of many dentists of this state. Under the guise of dental missionaries certain so-called instructors collected fees from dentists for educational services, when as a matter of fact the object of the expedition was to stimulate the sale of a certain apparatus, the distribution of which among dentists was not up to that time sufficiently satisfactory to the financial ambition of its manufacturer.

An inquiry into the qualifications, pedagogic and professional, of these self-appointed post-graduate teachers, in most instances reveals nothing that by any stretch of the imagination could be construed in the light of a license to impart instruction to under-graduates, to say nothing of graduates of years of standing. Any one who is not versed in all the scientific principles which constitute the foundation of technical procedures that are to be taught at these psuedo-educational courses should be barred by moral or legal force from occupying a teacher's platform, and as most of these so-called instructors are bearers of the badge of partial and therefore dangerous culture, their elimination from the educational field will be warmly acclaimed wherever and by whomsoever dentistry is held on a plane of strict professionalism.

A technical procedure, a surgical intervention, a new method of curing (?) pyorrhea, or what not—based upon the evidence of one man's word without other substantiating proofs, clinical, radiographic, bacteriologic, etc., and all of it the outcome of an intelligent analysis of the information available on the main and correlated subjects, can not be accepted in the light in which they are presented. The perpetrators of such teachings fulfill satisfactorily the requirements of professional piracy.

Post-graduate instruction, when it emanates from men who because of their experience, knowledge and analytical trend of mind have rightfully gained the title of dental teachers, is of undoubted value and adds to the assets of dentistry. When given by those who find in the prospects of material gain the only pretext for pedagogic self-appointment and who are men of only mediocre cultural or scientific attainments it drains the resources of dentistry and sprinkles its escutcheon with one of the most pernicious brands of empiricism.—Editorial in *Pacific Gazette.*

